# Zinc oxide (ZnO) hybrid metasurfaces exhibiting broadly tunable topological properties

**DOI:** 10.1515/nanoph-2022-0115

**Published:** 2022-06-10

**Authors:** Yuhao Wu, Sarah N. Chowdhury, Lei Kang, Soham S. Saha, Alexandra Boltasseva, Alexander V. Kildishev, Douglas H. Werner

**Affiliations:** Department of Electrical Engineering and Center for Nanoscale Science, The Pennsylvania State University, University Park, PA 16802, USA; Elmore Family School of Electrical and Computer Engineering, Purdue University, West Lafayette, IN 47907, USA; Birck Nanotechnology Center, Purdue University, West Lafayette, IN 47907, USA

**Keywords:** polarization synthesis, topological photonics, tunable metasurfaces

## Abstract

Extreme light confinement observed in periodic photonic structures, such as the vortex singularities in momentum (*k*) space, has been associated with their topological nature. Consequently, by exploiting and tuning their topological properties, optical metasurfaces have been demonstrated as an attractive platform for active photonics. However, given the fact that most active media under external excitations can only provide limited refractive index change, the potential advancements offered by the topological character of active metasurfaces have remained mostly unexplored. Zinc oxide (ZnO), which has recently exhibited optically-induced extraordinarily large permittivity modulations at visible and near-infrared frequencies, is an excellent active material for dynamic metasurfaces exhibiting strong tuning. This work demonstrates that a hybrid metasurface consisting of an array of ZnO nanodisks on a silver backplane displays broadly tunable topological properties. In particular, by performing *k*-space scattering simulations using measured pump-fluence-dependent material properties of ZnO, we study in detail the light reflection from the hybrid metasurface. Our results validate that the large *k*-space topology tuning of the metasurface can result in enormously strong polarization manipulation of near-infrared light in the vicinity of the topological features. The observed polarization switching effect is highly sensitive to the polarization and wavelength of an incident wave, owing to the symmetry and dispersion characteristics of the proposed system. Our study indicates that leveraging a combination of the extraordinary material properties and the *k*-space topology, hybrid metasurfaces based on ZnO may open new avenues for creating all-optical switchable metadevices.

## 1 Introduction

Artificial optical structures have facilitated enhanced light–matter interaction on a nanometer scale for comprehensive manipulation of light [1]. Evolving from metamaterials, the recently emerging metasurfaces based on single-layer or few-layer meta-atoms offer unprecedented flexibility in manipulating the intrinsic properties of optical waves, including phase, amplitude, polarization, angular momentum, etc. This has led to the demonstration of intriguing phenomena such as anomalous beam steering [2, 3], photonic spin hall effect [4], invisibility skin cloaks [5], metalensing [6], helicity-controlled SPP coupling [7, 8], etc. Moreover, in parallel to the explorations of metasurface-enabled optical phenomena, the development of active metasurfaces exhibiting unprecedented dynamic and reversibly tunable responses has attracted increasing attention in recent years [9]. There are two main reasons for this: first, in metasurfaces, the interaction between light and their planar architectures occurs in a subwavelength thickness, allowing engineers to efficiently introduce active materials that possess variable refractive indices into meta-atoms while circumventing complex three-dimensional (3D) fabrication; second, the ultrathin feature of metasurfaces significantly reduces the difficulties in regulating their responses to external excitations (such as laser pumping), resulting in excellent modulation dynamics.

The realization of active metasurfaces has been based on two primary strategies: first, hybridizing metals and active media; second, creating metasurfaces directly using meta-atoms composed of active materials. The property variation of an active material under external stimuli is a key factor in designing a superior active metasurface. In particular, the potential response modulation of an active metasurface is determined by a series of parameters associated with the property variation offered by the constituent active material, including an absolute refractive index change (in both real and imaginary parts), temporal dynamics, linearity, etc. In recent studies, various active media, such as phase-change materials [10–14], 2D materials [15–18], semiconductors [19–21], and so on, have been explored to enable dynamically tunable metasurfaces. However, examples of active materials which can offer ultrafast and large refractive index modulation in the visible and near-infrared (near-IR) remain rare.

Transparent conducting oxides (TCOs) have emerged as alternative plasmonic materials due to their tailorable optical properties [22] and low optical losses [23]. Their low process temperature, high performance, and fabrication flexibility have made them the ideal candidate for a plethora of applications, including solar cells [24], optoelectronic devices such as flat panel displays [25], light-emitting diodes [26], waveguide-integrated plasmonic modulators [27], and ultrafast all-optical switching [28–30]. Such high-speed optical modulation in TCOs can be easily achieved due to the significant variation of optical properties, with a relaxation time of the free hot carriers ranging from hundreds of picoseconds to sub-picoseconds [30–33]. Metal transparent oxides have been recently explored to dynamically tune the optical properties of metamaterials, which are governed by the free electrons in oxide electronic devices [34]. TCOs have a free carrier density on the order of 10^20^–10^21^ cm^−3^ resulting in a high DC-conductivity comparable to noble metals (∼10^22^ cm^−3^) with the bulk plasma frequency in the mid-infrared regime [34]. These properties make the materials transparent yet conductive above the plasma frequency enabling interesting tunable optical functionalities. Among different TCOs, low-loss, undoped zinc oxide (ZnO) has been utilized due to its well-established fabrication procedures, thickness uniformity, chemical stability, near-perfect absorption in multilayer structures [27], and detailed experimental studies of its steady-state optical properties with various dopants [35]. The performance of ZnO can be easily controlled by various methods like introducing dopants and varying the carrier concentration [29], heat treatment [36], controlling deposition parameters such as energy density, temperature, and multilayer stacking with various materials [23], adjusting the switching activation and electrode engineering [37].

We have recently demonstrated that silicon-nanostructure/gold-backplane hybrid metasurfaces exhibit nontrivial topological properties, which can facilitate polarization switching of near-infrared light by utilizing the dynamics of the photoexcited carriers in the silicon resonators [38]. However, due to the rather limited refractive index change silicon can provide below the damage threshold, the advantages offered by the topological nature of the system were only exploited to a small extent. The topology, including the curved boundary regions and the topological charges observed in the scattering properties in momentum (*k*) space, reflects the angular dispersion and polarization sensitivity of the interaction between light and hybrid metasurfaces. This work starts with analyzing the correlation between the eigenmode resonance and the scattering field. We show that the on-resonance iso-frequency contour dominates the scattering property of the proposed hybrid metasurfaces and highlights the topological feature in *k*-space.

We note that prior work by Saha et al. has demonstrated ZnO metasurfaces as ultra-fast reflection modulators through transient pumping of photoexcited carriers [30]. There, the authors excited dielectric zinc oxide with an interband optical pulse (266 nm), generating free carriers and causing the permittivity of the material to become more negative with its absorption increased via the Drude dispersion. The authors observed changes up to −3.6 in the real part of the dielectric permittivity at the technologically critical wavelength of 1600 nm. Such a large permittivity change enabled a broadband reflectance modulation of up to 70% in metal-backed oxide mirrors and metasurfaces. The 55% modulation at lower pump fluences was experimentally demonstrated. The metasurfaces and planar structures had picosecond-scale relaxation times.

Here, utilizing the relationship between pump fluence and carrier concentration presented in [30] we create a ZnO-based polarization modulator. The simulated ZnO disks were mapped by considering the fabrication imperfections that can change their overall predicted morphology and optical response [30]. By performing *k*-space scattering simulations using ZnO’s measured pump-fluence-dependent material properties, we study in detail the light reflection from a hybrid metasurface consisting of an array of ZnO nanodisks on top of a silver backplane. Our results show that optical excitation leads to a large-scale control over the topological property of the metasurface. This control results in enormously strong polarization manipulation of near-IR light in the vicinity of the *k*-space topological features.

Moreover, we verify that the observed polarization switching effect is highly sensitive to the polarization and wavelength of an incident wave, which can be attributed to the symmetry and dispersion characteristics of the proposed system. Our study also indicates that the *k*-space topology tuning of the metasurface, based on the extraordinarily large permittivity change in ZnO, opens many exciting opportunities for manipulating the intrinsic properties of visible and near-IR light. This is expected to lead to a new family of active metadevices capable of realizing sophisticated polarization synthesis and all-optical switching.

## 2 Results and discussion

**Figure 1:**
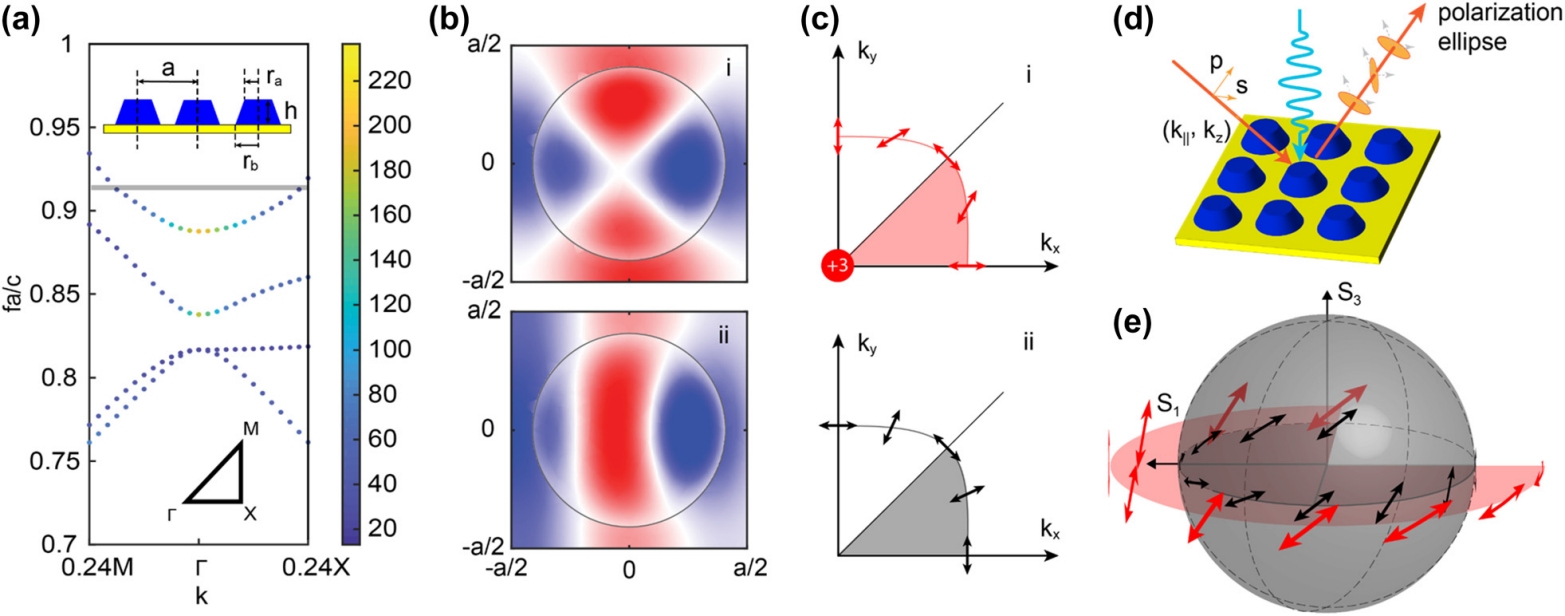
ZnO/Ag hybrid metasurfaces for all-optical manipulation of light polarization. (a) The band diagram of the ZnO/Ag hybrid metasurface made of a ZnO nanodisk array deposited on an optically thick Ag backplane. The color of the markers represents the corresponding radiative *Q*-factor. A side-view schematic of the hybrid metasurface is also presented as an inset: *r*_*a*_ = 270 nm, *r*_*b*_ = 450 nm, *h* = 180 nm, and square lattice with *a* = 1100 nm. (b) Eigenmode profile of *E*_*z*_ on the TE_4_ band, for in-plane wavevector *k*_‖_ parallel to the (i) ΓM direction, and (ii) ΓX direction, respectively. (c) On-resonance SOPs of the (i) eigenmodes, and (ii) scattering field when the system is illuminated by *s*-pol light. (d) Schematic of the hybrid metasurface for reflection polarization switching based on a photoexcited large permittivity change in the ZnO resonators. (e) Quasi-linear SOPs of the eigenmodes (red arrows) on the equator of a Poincare sphere when *k*_‖_ moves on the iso-frequency contour. The corresponding SOPs of the scattering field (black arrows) are also presented for comparison.

The proposed metasurfaces are composed of an array of frustum-shaped ZnO nanodisks (top radius, *r*_*a*_ = 270 nm, bottom radius, *r*_*b*_ = 450 nm, and height *h* = 180 nm) on top of an optically thick silver substrate, as shown in Figure 1(d). The unit cell is arranged in a two-dimensional square lattice with a lattice constant of *a* = 1100 nm. The shape of the ZnO nanodisks was determined by taking into account the sidewall effect observed in previous studies of ZnO nanostructures [30]. At the same time, it has been known that the tilted sidewall significantly influences the quality (*Q*) factor of the resonant modes supported by the nanodisks. To understand the underlying mechanism of the wavevector (*k*) dependent far-field response of the proposed metasurface, the band structure and the corresponding eigenmodes of the periodic structure are analyzed theoretically and numerically. Figure 1(a) depicts a color-coded band diagram of the metasurfaces. The color of the markers represents the radiative *Q*-factor of the corresponding eigenmodes. Figure 1(b) illustrates the electric field profiles (Re(*E*_*z*_)) of the two iso-frequency (*ω*_0_ = 0.919 × 2π*c*/*a*, grey line in Figure 1(a)) eigenmodes on the fourth TE (TE_4_) band in the high-symmetry (ΓM and ΓX) directions. The *s*-polarized (*s*-pol) and *p*-polarized (*p*-pol) distributions can be identified for the eigenmodes along the ΓM and ΓX directions, respectively. According to the temporal coupling mode theory (TCMT) [39], these observations correspond to a zero-valued *p*-pol (*s*-pol) coupling coefficient, i.e., |*d*_*s*_|(|*d*_*p*_|) = 0, and a maximum-valued *s*-pol (*p*-pol) coupling coefficient, i.e., |*d*_*p*_|(|*d*_*s*_|) = (2*γ*)^1/2^, where *γ* is the radiation loss of the resonance modes. In (i) of Figure 1(c), the iso-frequency contour at *ω*_0_ (red) is plotted inside the 1st quarter of the reciprocal space. We note that, on the 3D band surface of the TE_4_ mode, the corresponding states of polarization (SOPs) of the in-plane eigenmodes experience a 3*π*/4 rotation from a *p*-pol (at ΓX) state to an *s*-pol (ΓM) state, which constitutes a topology of winding number +3 around the polarization singularities at the Γ point.

The SOPs of the resonance can be represented by a parametric vector as indicated in Eq. (1), where *θ*_*s*_ and *θ*_*p*_ denote the phase of the *s*-pol and *p*-pol resonance modes, respectively. The parameters, i.e., *α*, *β* are governed by the energy conservation rule (*d*_*p*_^2^ + *d*_*s*_^2^ *=* 2*γ*), *α* + *β* = 2. Therefore, the rotation of the resonance polarization can be theoretically regarded as an increase (decrease) in the parameter *α* (*β*) from 0 (2) to 2 (0). Note that the corresponding eigen resonance modes are quasi-linearly polarized because the phase difference between *θ*_*s*_ and *θ*_*p*_ is negligible [40]. Consequently, the SOPs of the reflection wave along the iso-frequency contour can be obtained from the TCMT scattering matrix (Eq. (2)) [38, 41], in which *ω*_0_ is the resonance frequency, *σ*_*z*_ is the Pauli matrix, $C=\left(\begin{array}{cc} C_{\text{ss}} & 0\\ 0 & C_{\text{pp}} \end{array}\right)$ is the background reflection coefficient of the resonance system (for a perfect electric conductor (PEC) backed system, *C*_ss_ = *C*_pp_ = 1), and *γ* is the radiation loss rate of the system. As shown in (ii) of Figure 1(c), when an s-pol wave illuminates the metasurface, the quasi-linear SOPs of the scattering wave also experience a 3π/4 polarization rotation along the iso-frequency contour (black curve). On the iso-frequency contour, an intermediate mode (|*d*_*p*_| = |*d*_*s*_ *|* = (*γ*)^1/2^) of complete *s*-to-*p* conversion, reveals itself as two oppositely signed topological charges in the vectorized scattering field in the first quadrant [38, 41]. Apparently, the rotation of the SOPs and the topological charges in the vectorized scattering field originated from the winding topology of the TE modes around the Γ point.

(1) $$\begin{array}{c} d=\left(\begin{array}{c} d_{s}\\ d_{p} \end{array}\right)=\left(\begin{array}{c} \sqrt{\alpha \gamma }{\text{e}}^{\text{i}\theta_{s}}\\ \sqrt{\beta \gamma }{\text{e}}^{\text{i}\theta_{p}} \end{array}\right) \end{array}$$

(2) $$\begin{array}{ll} r & =\left(\begin{array}{cc} r_{\text{ss}} & r_{\text{ps}}\\ r_{\text{sp}} & r_{\text{pp}} \end{array}\right)=-\sigma_{z}\left\{I-\frac{dd^{†}}{\text{i}\left(\omega_{0}-\omega \right)+\gamma }\right\}C\\ & =\left(\begin{array}{cc} \alpha -1 & \sqrt{\alpha \beta }{\text{e}}^{\text{i}\theta_{s}-\theta_{p}}\\ -\sqrt{\alpha \beta }{\text{e}}^{\text{i}\theta_{p}-\theta_{s}} & 1-\beta \end{array}\right) \end{array}$$

To better illustrate the polarization of the reflected wave along the on-resonance iso-frequency contour, the corresponding SOPs are mapped onto a Poincare sphere, as illustrated in Figure 1(e). The quasilinear SOPs are identified on the 3/4 equator of the Poincare sphere. A close inspection shows that the on-resonance iso-frequency contour indicates the potential of the metasurface for polarization synthesis upon reflection and defines a phase transition boundary of the SOPs of the scattering field (see detailed in the Supplementary Information). We note that the observed rotation of the SOPs is attributed to the symmetry and topological nature of the structure [38, 40, 41]. Therefore, a similar methodology has also been implemented to analyze the metasurfaces with C_3_-symmetry, as shown in Section 3 of the Supplementary Information. With three scattering topological charges along the on-resonance contour in the first quadrant, a more rapid rotation of the quasilinear SOPs is observed in the C_3_-symmetric system.

**Figure 2:**
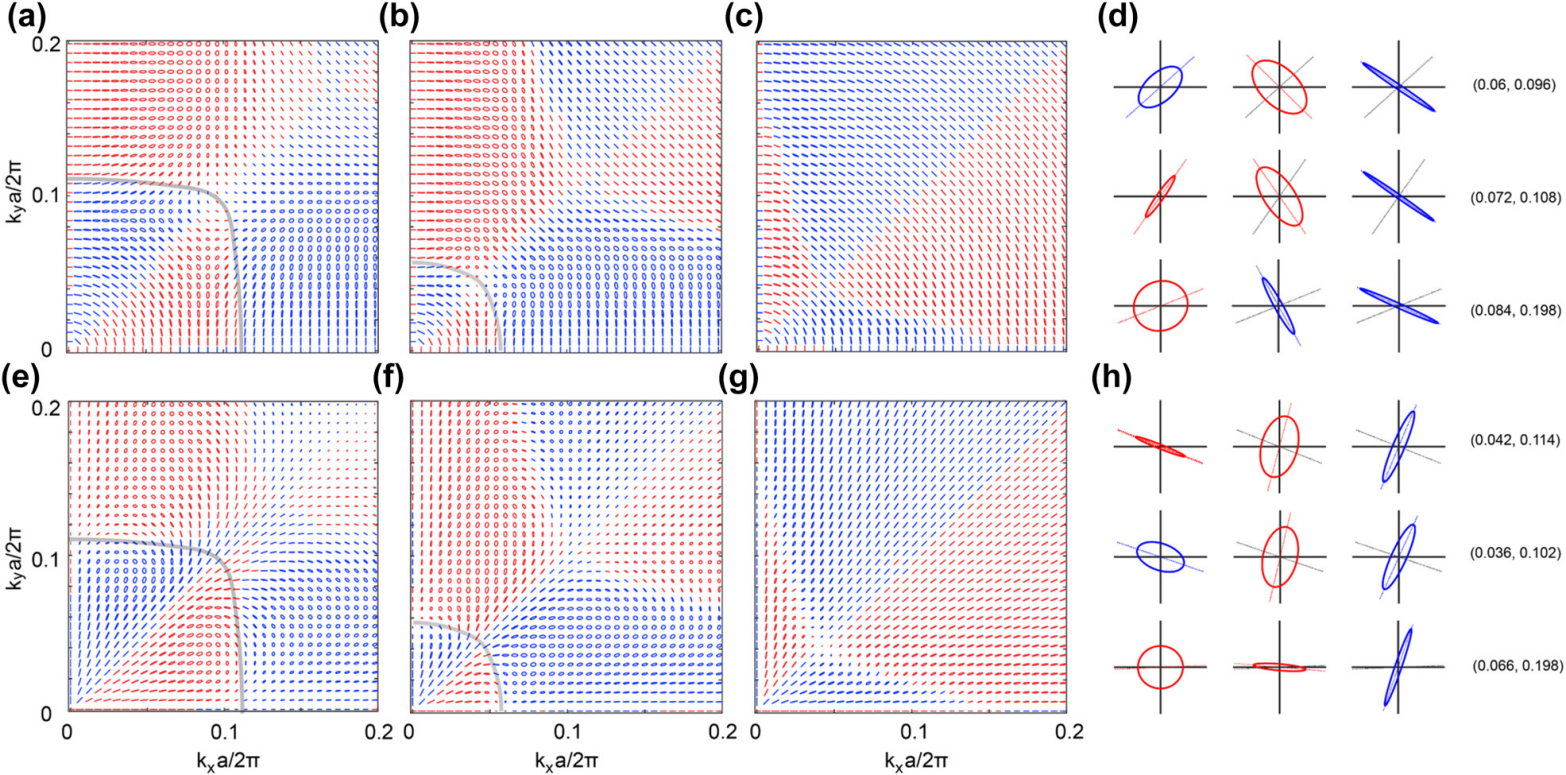
Polarization synthesis based on the ZnO/Ag hybrid meta-surface. Polarization ellipses as a function of wavevectors for *s*-pol incident light at *ω*_0_ = 0.919 × 2π*c/a* (*λ*_0_ = 1197 nm), when the metasurface is (a) in its static state, and under an excitation of 266 nm light at a pump fluence of (b) 1.31 mJ/cm^2^ (ΔRe(ε) = −0.326) and (c) 2.63 mJ/cm^2^ (ΔRe(ε) = −0.949). (d) Polarization ellipses across the three states at (*k*_*x*_, *k*_*y*_) = (0.06, 0.096), (0.072, 0.108), and (0.084, 0.198). (e)–(g) The same as panels (a)–(c), but for *p*-pol illumination. (h) Same as panel (d) but for *p*-pol illumination at (*k*_*x*_, *k*_*y*_) = (0.042, 0.114), (0.036, 0.102), and (0.066, 0.198). In all panels, the red (blue) ellipses denote right(left)-handed polarization. For panel (d) and (h), the dashed line in each panel denotes the main axis of the ellipse.

To exploit the polarization synthesis potential offered by the proposed metasurfaces, the polarization states of the reflection field as a function of the wavevector of the *s*-pol incident light are numerically computed at *ω*_0_. Figure 2(a)–(c) illustrate the corresponding polarization ellipses of the reflection wave when the metasurface is in its static state (without optical excitation) and when it is optically excited by 266 nm light at a pump fluence of 1.31 mJ/cm^2^ and 2.63 mJ/cm^2^. It is important to note that the pump fluences used in our study are rather moderate compared with those used to achieve the maximum permittivity change in the previously reported studies [30]. Each ellipse is plotted in a local coordinate system on the *x-y* basis. The red (blue) color indicates the right (left) handedness of the polarization, while the dimension of the ellipses denotes the reflection magnitude. The on-resonance iso-frequency contour is also presented as a grey curve in Figure 2(a) and (b), which defines the boundary of the polarization transition (between left-handed and right-handed) in *k*-space. Importantly, along the contour, the SOPs of the scattering field are quasi-linearly polarized and indicate a rapid polarization rotation. Abundant polarization states are achieved in the subdomains adjacent to the iso-frequency contour. As demonstrated previously [40], these polarization-abundant domains contain all possible polarization states across the Poincare sphere surface.

Furthermore, the measured results have shown that a 266 nm optical excitation at a fluence of 1.31 mJ/cm^2^ can lead to a permittivity (real part) decrease of 0.326 [30], which can result in a significant blueshift of the TE_4_ band of interest. Accordingly, as shown in Figure 2(b), the iso-frequency contour at *ω*_0_ will shrink towards the Γ point, leading to a significant change in SOPs in *k*-space. Moreover, as shown in Figure 2(c), for the excitation at a fluence of 2.63 mJ/cm^2^, which corresponds to a permittivity (real part) decrease of 0.949 [30], the resonance (iso-frequency contour) vanishes at frequency *ω*_0_. Correspondingly, this occurs along with the drift and annihilation of the topological charges (see Figure S3 in the Supplementary Information). Accordingly, the polarization-abundant domains disappear, and the scattering field will be purely *s*-pol across the *k*-space of interest. The collapse of the polarization-abundant domains is associated with the annihilation of topological charges observed in the vector field of the scattering coefficient [38, 41]. Figure 2(e)–(g) presents the *k*-dependent polarization ellipses for a *p*-pol illumination at *ω*_0_. A similar movement of the iso-frequency contour, which highlights the complete polarization conversion effect (as well as the polarization-abundant domains), can be identified. Results shown in Figure 2(a)–(d) and (e)–(g) indicate the great potential of the proposed metasurfaces for polarization synthesis upon reflection, and the optical excitation enabled polarization switching of light.

In Figure 2(d), the SOPs at three discrete wavevectors, *k*_‖_ = (0.06, 0.096) × 2*πc*/*a*, (0.072, 0.108) × 2*πc*/*a*, and (0.084, 0.198) × 2*πc*/*a* are illustrated to better showcase the polarization control capability of the proposed platform under *s*-pol illumination. From left to right, the three polarization ellipses in each row correspond to the three states, i.e., static state, and the optical excitations at fluences of 1.31 mJ/cm^2^ and 2.63 mJ/cm^2^ discussed above. The dashed line, which represents the main axis of the polarization ellipse, is depicted to illustrate the polarization rotation angle, *ψ*, relative to the initial (static) state. The maximum polarization rotation angles of 86.14° and 89.64° are observed in the first two rows, respectively. These results suggest that by selecting the wavevector of the incident wave, almost perfect polarization orthogonality can be achieved for the reflection field using a moderate optical excitation. On the other hand, for *k*_‖_ = (0.084, 0.198) × 2π*c*/*a*, optical excitations lead to a large ellipticity variation from a quasicircular polarization at the static state to a quasilinear polarization. Similarly, Figure 2(h) shows the reflection polarization states under a *p*-pol illumination at three wavevectors, *k*_‖_ = (0.042, 0.114) × 2*πc*/*a*, (0.036, 0.102) × 2*πc*/*a*, and (0.066, 0.198) × 2*πc*/*a*. A similar optical excitation enabled polarization orthogonality is observed in the first two rows. In contrast, the maximal ellipticity modulation from circular polarization (CP) to linear polarization (LP) is identified in the third row.

In principle, the maximal polarization rotation of the scattering field originates from the corresponding rotation of the quasi-linear in-plane resonances along the iso-frequency contour. On the other hand, the polarization-abundant domains nearby yield a comprehensive coverage of the Poincare sphere surface from the LP equator to the two CP poles. Therefore, for both *s*-pol and *p*-pol incident light, the maximal polarization rotation occurs at wavevectors around the iso-frequency contour (grey curves in Figure 2(a)–(b) and (e)–(f)), which corresponds to the equator of a Poincare sphere. On the contrary, the maximal ellipticity modulation is achieved around the center of the polarization-abundant domain, which corresponds to the two poles on a Poincare sphere.

**Figure 3:**
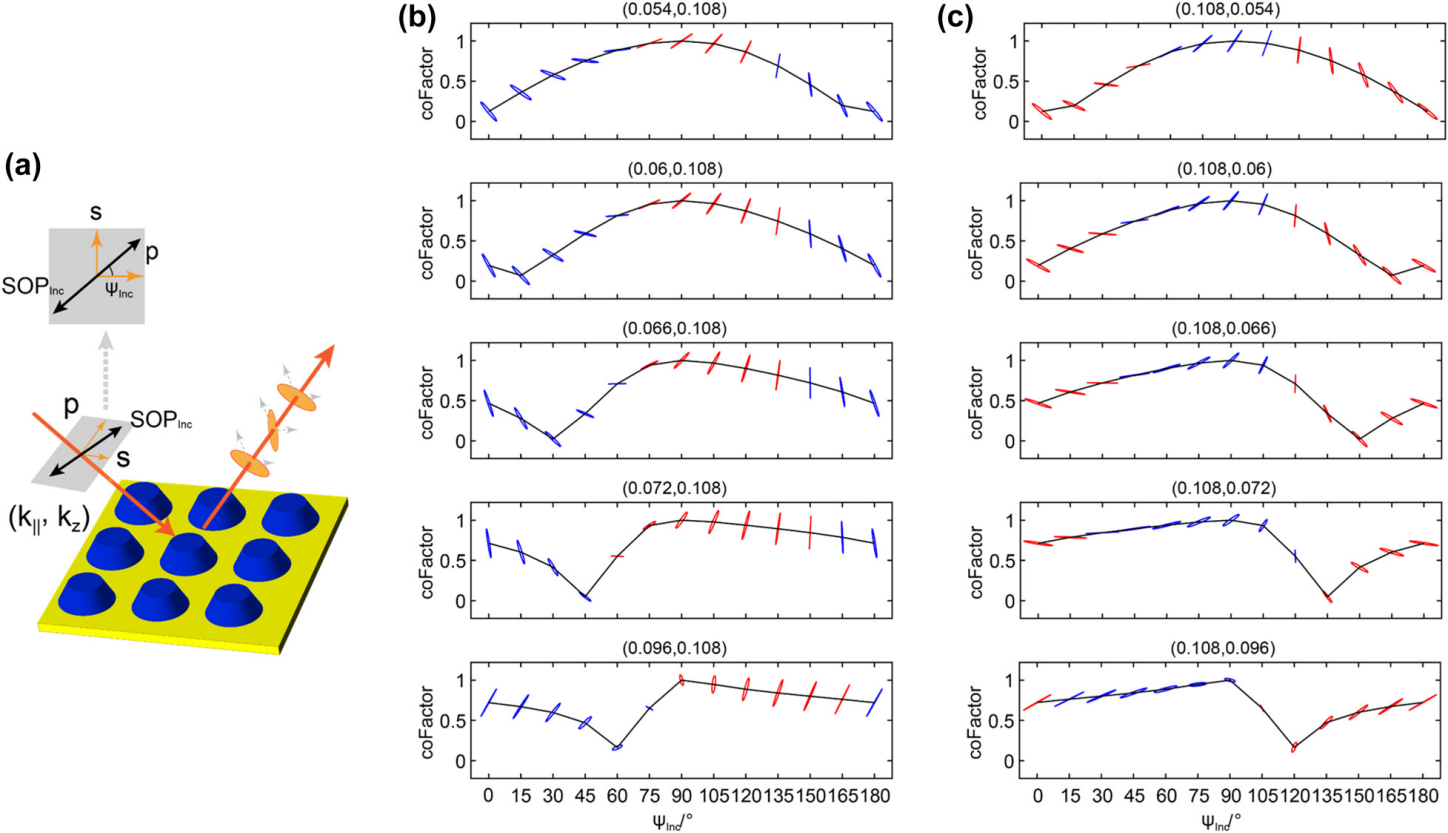
Reflection polarization as a function of the incident polarization. (a) Schematic of the ZnO/Ag metasurface under illumination of a linearly polarized wave with a polarization rotation angle *ψ*_Inc_. (b)–(c) Correlation factor (*coFactor*) as a function of the incident rotation angle *ψ*_Inc_, at a series of discrete wavevectors. The polarization ellipses of the corresponding reflected wave are also presented.

To further demonstrate the versatility of the proposed metasurface for polarization modulation, the static reflection behavior of the device under the illumination of an arbitrary linearly polarized wave at a series of wavevectors (defined by a polarization angle *ψ*_Inc_ shown in Figure 3(a)) is summarized in Figure 3. As presented in Eq. (3), the SOP modulation upon reflection is quantified by a correlation factor (*coFactor*), which is defined as a normalized inner product between the reflection field of *s*-pol incident light (*ψ*_Inc_ = 90°) and that of LP-incident light (arbitrary *ψ*_Inc_). In Eq. (3), the *s*-component of the complex reflection coefficient is denoted as *r*_*s*,*ψ*_, and the *p*-component as *r*_*p*,*ψ*_.

(3) $$\begin{array}{ll} \text{c}\text{o}\text{F}\text{a}\text{c}\text{t}\text{o}\text{r} & =\frac{1}{\sqrt{{r_{s,90}}^{2}+{r_{p,90}}^{2}}}\left(\begin{array}{c} r_{s,90}\\ r_{p,90} \end{array}\right)\\ & \;\times \frac{1}{\sqrt{{r_{s,\psi_{\text{Inc}}}}^{2}+{r_{p,\psi_{\text{Inc}}}}^{2}}}\left(\begin{array}{c} r_{s,\psi_{\text{Inc}}}\\ r_{p,\psi_{\text{Inc}}} \end{array}\right) \end{array}$$

In particular, the value of *coFactor* will be zero when the two field vectors are orthogonal to each other, while it will be unity if the two field vectors are parallel to each other. Thus, *coFactor* can be used to represent the degree of polarization angle modulation upon reflection. In Figure 3(b), *coFactor* is plotted as a function of the incident rotation angle at five discrete wavevectors, in which the highest polarization orthogonality (between the incident and reflected waves) is achieved at *ψ*_Inc_ = 0°, 15°, 30°, 45°, and 60°, respectively. The corresponding polarization ellipses of the reflection field are also presented. It can be seen that for incident light at different *ψ*_Inc_ an appropriate selection of *k*_‖_ can lead to perfect polarization orthogonality.

Interestingly, near-zero values of *coFactor* always correspond to the wavevectors on the iso-frequency contour with a concomitant quasilinear polarization of the reflection field. These observations offer another explicit demonstration of the polarization modulation potential of the proposed metasurface. Comparing the results with Figure 3(b) and (c) it shows another five wavevectors, which are symmetric with respect to ΓM. This feature is indicative of the C_4_ rotational symmetry of the metasurfaces.

**Figure 4:**
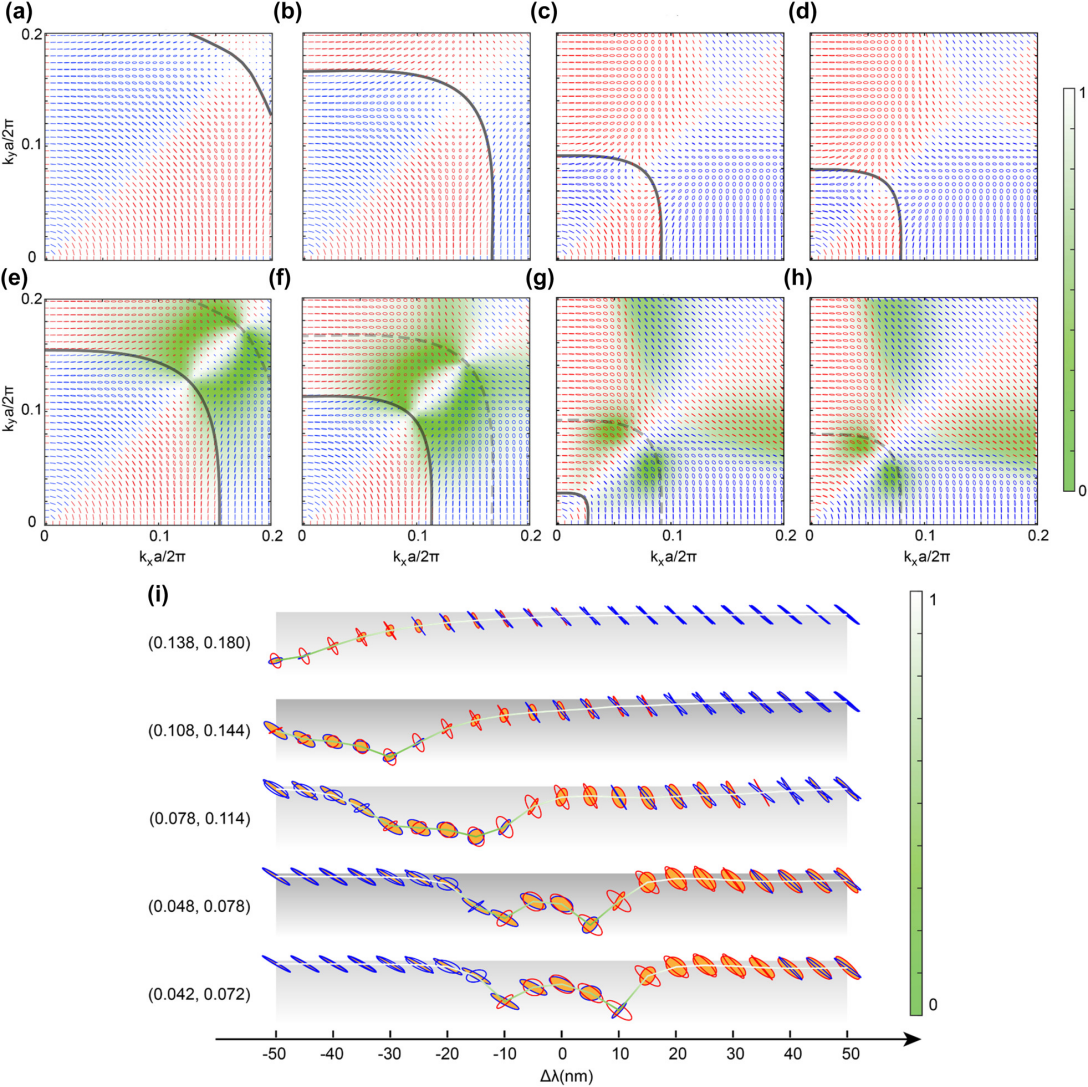
Frequency detuning enabled polarization modulation. (a)–(d) Static polarization ellipses as a function of wavevectors of the *s*-pol incident light at *λ* = 1150 nm, 1170 nm, 1205 nm, and 1210 nm. (e)–(h) Same as panels (a)–(d) but for an optical excitation of fluence of 1.31 mJ/cm^2^ (ΔRe(*ε*) = −0.362). The color-coded background represents the correlation factor between the polarization states at static and optically excited states at the corresponding wavelengths. (i) The correlation factor at selected wavevectors as a function of the wavelength. The corresponding polarization ellipses at the static state (orange-filled) and the excited state (open) are also presented, where the red (blue) color of the ellipses represents the right (left)-handedness.

The resonant nature of the proposed metasurface suggests that the reflection response of the system is highly sensitive to the wavelength of the incident light. In the discussion above, we consider a fixed incident frequency *ω*_0_ = 0.919 × 2*πc*/*a* (*λ*_0_ = 1197 nm). To provide a complete picture of the optical excitation enabled response tuning of the ZnO-based metasurface systems, the reflection spectra under different pumping fluences are simulated and summarized in Section 2 of the Supplementary Information. Here, to be specific, Figure 4(a)–(d) illustrate the *k*-dependent reflection polarization ellipses when the metasurface is illuminated by *s*-pol light at four additional wavelengths near *λ*_0_, i.e., *λ* = 1150 nm, 1170 nm, 1205 nm, and 1210 nm. A color-coded background indicates the reflection field’s correlation factor (*coFactor*) at *λ* by referencing it to that at *λ*_0_. The iso-frequency contours for each wavelength *λ* are depicted on the *k*-space map (black curve). At the static state (no optical excitation), the dispersion relation of the metasurface remains unchanged. Thus, a detuning of the incident wavelength corresponds to a movement of the iso-frequency cut on the 3D band structure of the metasurface. Since the TE_4_ band has an approximately parabolic shape that opens upwards, the iso-frequency contour will shrink towards the Γ-point as the frequency redshifts (wavelength increases). The trend can be seen from the movement of contours (black) shown in Figure 4(a)–(c), while when *λ* further increases to 1210 nm (Figure 4(d)), the iso-frequency contour vanishes, indicating that the system is off-resonance. On the other hand, the frequency detuning can also lead to dramatic changes in the polarization-abundant domains. The observed wavelength sensitivity of the system originates from the high-*Q* resonance supported by the metasurface.

To illustrate the frequency detuning enabled flexibility in active polarization modulation, Figure 4(e)–(h) show the corresponding reflection polarization ellipses when the metasurface is under an optical excitation at a fluence of 1.31 mJ/cm^2^. The color-coded background represents the correlation factor (*coFactor*) between the polarization states in the static and optically excited states at the corresponding wavelengths. The isofrequency contour at the optically excited state is plotted in each map as the black curve, while that in the static state as the dashed grey curve. As mentioned in the discussion above, the optical excitation used may lead to a blueshift of the TE_4_ band. Consequently, as confirmed by Figure 4(e)–(g), compared with that in the static state (dashed grey curve), the optical excitation causes the isofrequency contours at *λ* (black curve) to move closer to the Γ-point. Meanwhile, the iso-frequency contour vanishes in Figure 4(h), which indicates that the optical excitation results in the complete off-resonance state of the system in the *k*-space is of interest. Furthermore, it can be seen from Figure 4(e) and (f) that strong polarization modulation can be realized around the *k*-space region defined by the isofrequency contours of the static and photoexcited states (the black and grey dashed line), where the lowest *coFactor* (green area) can be seen. Moreover, in the scenarios where the isofrequency contours approach the Γ-point (Figure 4(g)) or completely vanish (Figure 4(h)), strong polarization modulation occurs in the *k*-space regions adjacent to the isofrequency contour of the static state.

To clearly show frequency detuning enabled flexibility in the polarization modulation, the polarization ellipses at five wavevectors as a function of the wavelength in the static and optically excited states are shown in Figure 4(i). The corresponding dispersion of the correlation factor reflects the polarization modulation depth. Note that the studied wavevectors correspond to the most pronounced polarization modulation induced by the optical excitation. Moreover, the correlation factor spectra at each wavevector are color-coded to better represent their extracted value. At each wavelength, the corresponding polarization ellipses in the static state (orange-filled ellipse) and the excited state (open ellipse) are also presented. Figure 4(i) unambiguously demonstrates the frequency detuning enabled polarization modulation offered by the proposed system supporting high-*Q* resonances.

## 3 Conclusions

In summary, we have demonstrated a ZnO nanodisk-based hybrid metasurface for all-optical switching of near-infrared light polarization. The observed strong polarization modulations are based on the pronounced topological property tuning of the metasurface, which is made possible by the optically induced extraordinarily large permittivity modulations in ZnO. In particular, our study shows that the dramatic variation of the reflection polarization originates from the high-*Q* quasilinear resonance modes supported by the metasurface, which are highly sensitive to the in-plane wavevectors. As a result, on-resonance iso-frequency contours dominate the scattering properties of the proposed metasurfaces and suggest the potential regions in *k*-space where the most pronounced polarization modulation occurs. Given the ultrafast dynamics of ZnO reported in previous studies, we envision that the proposed ZnO hybrid metasurfaces can be employed to facilitate strong and ultrafast modulation of the polarization of light at visible and near-infrared frequencies.

## Supplementary Material

Supplementary Material Details
