# Supplementary material for: Zinc oxide (ZnO) hybrid metasurfaces exhibiting broadly tunable topological properties
Source: Nanophotonics. 2022 Jun 10;11(17):3933–42. doi: 10.1515/nanoph-2022-0115 (PMC11501124; doi:10.1515/nanoph-2022-0115)
Supplement: Supplementary file 1 — Supplementary Material Details [file j_nanoph-2022-0115_suppl.docx]

Supplementary Information

Zinc oxide (ZnO) hybrid metasurfaces exhibiting broadly tunable topological properties

Yuhao Wu^1,†^, Sarah N. Chowdhury^2,3,†^, Lei Kang^1^, Soham S. Saha^2,3^, Alexandra Boltasseva^2,3,^*, Alexander V. Kildishev^2,3,^*, and Douglas H. Werner^1,^*

^1^ Department of Electrical Engineering and Center for Nanoscale Science,

The Pennsylvania State University, University Park, Pennsylvania 16802, United States

^2^ Elmore Family School of Electrical and Computer Engineering, Purdue University, West Lafayette, IN 47907, USA

^3^ Birck Nanotechnology Center, Purdue University, West Lafayette, IN 47907, USA

^†^ Y. W. and S. N. C. contributed equally to this work.

**S1. Temporal coupling mode analysis of the states of polarization (SOPs)**

According to previous studies,^1–3^ the scattering matrix of a reflective system with C_4_ symmetry can be explicitly expressed as Eq. (S1),

$$\begin{aligned} r=\left( \begin{matrix} r_{ss} & r_{ps} \\ r_{sp} & r_{pp} \end{matrix} \right)={-\sigma}_{z}\left\{ I-\frac{d_{+}d_{+}^{\dagger}}{i\left( \omega_{0}-\omega\right)+\delta+\gamma} \right\}C_{+}={-\sigma}_{z}\left\{ I-\frac{1}{i\left( \omega_{0}-\omega\right)+\gamma}\left( \begin{matrix} d_{s}^{2} & d_{s}d_{p}^{*} \\ d_{p}d_{s}^{*} & d_{p}^{2} \end{matrix} \right) \right\} \#\left( S1 \right) \end{aligned}$$

$$\begin{aligned} d=\left( \begin{matrix} d_{s} \\ d_{p} \end{matrix} \right)=\left( \begin{matrix} \sqrt{\alpha\gamma}e^{i\theta_{s}} \\ \sqrt{\beta\gamma}e^{i\theta_{p}} \end{matrix} \right) \#\left( S2 \right) \end{aligned}$$

where *I* is the identity matrix, *d* is the coupling coefficient vector of the in-plane resonance, *ω* is the operating frequency, *ω*_0_ is the resonance frequency, $\sigma_{z}=\mathrm{diag}\left( 1,-1 \right)$is the Pauli matrix, $C=\mathrm{diag}\left( C_{ss},C_{pp} \right)$ is the background reflection coefficient of the resonance system (for a perfect electric conductor (PEC) backed system, *C*_ss_ *= C*_pp_ *=* 1), and *γ* and δ are the radiation loss rate and material loss rate of the system, respectively. The material loss is smaller than the radiation loss by orders, which can therefore be neglected in the topology study.

The SOPs of the eigenmodes can be formulated into a parametric vector as shown by Eq. (S2), in which *θ_s_* and *θ_p_* denote the phase of the *s*-pol and *p*-pol resonance modes, respectively. The parameters, *i.e.*, *α*, *β* are governed by the energy conservation rule $\left\| d \right\|\to\alpha+\beta=2$. Following this methodology, the SOPs of the on-resonance modes and the scattering field reflected out can be studied parametrically. The (*s-*pol) eigenmodes with the odd symmetry along the ΓM direction, have a polarization vector given as $\sqrt{2\gamma}\left( \begin{matrix} e^{i\theta_{s}} \\ 0 \end{matrix} \right)$, while the (*p-*pol) eigenmodes with the even distribution along the ΓX direction have an SOP vector $\sqrt{2\gamma}\left( \begin{matrix} 0 \\ e^{i\theta_{p}} \end{matrix} \right)$. By substituting these two polarization vectors into Eq. (S1), the corresponding scattering matrix *r* will be of the form $-I$ and $I$. Therefore, a polarization-preserved reflection will be observed upon *s-*pol illumination along these two directions.

On the other hand, if the two parameters are equal, *i.e.*, *α =* *β* = 1, the SOP vector will be $\sqrt{\gamma}\left( \begin{matrix} e^{i\theta_{s}} \\ e^{i\theta_{p}} \end{matrix} \right)$, which leads to a scattering matrix given by ${i\sigma}_{y}e^{i{(\theta}_{s}-\theta_{p})}$, with $\sigma_{y}$ being another Pauli matrix. At this specific wavevector, the reflection process is transformed into a complete polarization conversion, where a topological singularity of the scattering field can be identified on the *k-*space map.^2,3^ The SOPs of the on-resonance mode given in Eq. (S2) can be substituted into Eq. (S1) to yield the scattering matrix represented by Eq. (S3), which can be utilized to derive the polarization states of the reflection field. Here, the phase difference between the *s-*pol component and the *p-*pol component, *θ_s_* − *θ_p_* is denoted as Δ in the equation (usually assumed to be zero). Therefore, the corresponding eigen-resonance modes are quasi-linearly polarized. As the parameters *α* (*β*) increases (decreases) from 0 (2) to 2 (0), the quasi-linear polarization of the in-plane eigenmodes experience a 3π/4 rotation from a *p*-pol (at ΓX) state to an *s*-pol (ΓM) state. In Fig. S1(a), the rotation of the resonance polarization (double-headed red arrows) from ΓX to ΓM is depicted along the on-resonance iso-frequency contour (pink curve) by dividing the half quarter into five characteristic ranges.

$$\begin{aligned} r={-\sigma}_{z}\left\{ I-\frac{1}{\gamma}\left( \begin{matrix} \alpha\gamma& \sqrt{\alpha\beta}\gamma e^{i{(\theta}_{s}-\theta_{p})} \\ \sqrt{\alpha\beta}\gamma e^{i{(\theta}_{s}-\theta_{p})} & \beta\gamma\end{matrix} \right) \right\}=\left( \begin{matrix} \alpha-1 & {\sqrt{\alpha\beta}e}^{i\Delta} \\ {-\sqrt{\alpha\beta}e}^{-i\Delta} & 1-\beta\end{matrix} \right) \#\left( S3 \right) \end{aligned}$$

Following the parametric definition of each characteristic range, the quasi-linear *r-*field vector (double-headed black arrows) at each range upon a *s-*pol illumination is derived explicitly and presented along the same on-resonance iso-frequency contour in Fig. S1(b). Correspondingly, they also evolve through a 3π/4 rotation.


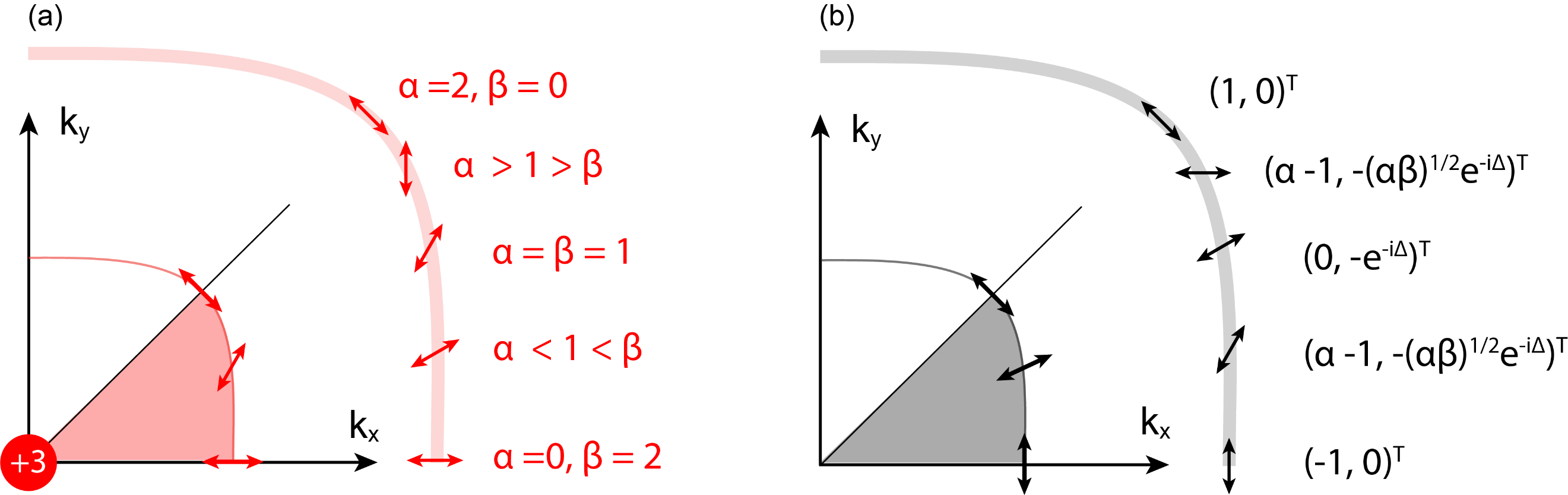


Fig. S1. Representative on-resonance SOPs of the eigenmodes (a), and scattering field (b) when the system is illuminated by *s*-pol light. The SOPs are selected at five representative parametric ranges, from ΓX to ΓM.

For the slightly off-resonance cases, Eq. (S3) can be represented as Eq. (S4) by adding an off-resonance imaginary term, *i*(*ω_0_ − ω*) to the denominator. Since the phase difference Δ between the *s-*pol resonance and *p-*pol resonance is negligible, it is excluded for simplicity in the following derivations. By defining a relationship between the frequency deviation and the radiation loss as *ω_0_ − ω = εγ*, the scattering matrix can be reformulated accordingly. For *s-*pol incident light, the reflection field can be expressed as Eq. (S5). As *ω_0_* deviates from the incident frequency *ω*, a deviation-related phase term will be embedded into the scattering field vector. This may change the polarization states from an on-resonance (*ε = 0*) linear polarization to an off-resonance elliptical polarization or even circular polarization. Therefore, the polarization abundant domains discussed in the main text occur around the on-resonance iso-frequency contour. However, for a more significant detuning (*ε >> 1*), the amplitude decay, 1/(*ε^2^+1*)^1/2^, will result in a reflection field that is purely *s-*pol light.

Furthermore, for a detuning in which the sign of *ε* changes, the scattering polarization states will transition from left-handedness (LH) to right-handedness (RH). Therefore, in the main text, the red (RH)-to-blue (LH) transition boundary observed in the *k-*space polarization ellipse map indicates the on-resonance iso-frequency contour, which corresponds to the polarization rotation of the quasi-linearly polarized modes.

$$\begin{aligned} r={-\sigma}_{z}\left\{ I-\frac{\gamma}{i\left( \omega_{0}-\omega\right)+\gamma}\left( \begin{matrix} \alpha& \sqrt{\alpha\beta} \\ \sqrt{\alpha\beta} & \beta\end{matrix} \right) \right\}={-\sigma}_{z}\left\{ I-\frac{1}{i\varepsilon+1}\left( \begin{matrix} \alpha& \sqrt{\alpha\beta} \\ -\sqrt{\alpha\beta} & \beta\end{matrix} \right) \right\}=\left( \begin{matrix} \frac{1}{i\varepsilon+1}\alpha-1 & \frac{1}{i\varepsilon+1}\sqrt{\alpha\beta} \\ -\frac{1}{i\varepsilon+1}\sqrt{\alpha\beta} & 1-\frac{1}{i\varepsilon+1}\beta\end{matrix} \right)\#\left( S4 \right) \end{aligned}$$

$$\begin{aligned} r\left( \begin{matrix} 1 \\ 0 \end{matrix} \right)= \left( \begin{matrix} \frac{1-i\varepsilon}{\varepsilon^{2}+1}\alpha-1 \\ \frac{i\varepsilon-1}{\varepsilon^{2}+1}\sqrt{\alpha\beta} \end{matrix} \right) \#\left( S5 \right) \end{aligned}$$

**S2. Reflection spectrum and the topological properties of the scattering field**

Figure S2 summarizes the optically active reflection spectra of two types of metasurfaces; ZnO/ Sapphire/Ag (*a* = 1100 nm, frustum-shaped nanodisk) and ZnO/PEC (*a* = 1000 nm, cylinder-shaped nanodisk). A notable blue-shift of the *s-*to-*p* polarization conversion peak is obs­­erved in both systems as the pumping fluence increases. Meanwhile, compared with the system with an idealized PEC-substrate, the resonance broadening effect is also observed in the Ag substrate system, which corresponds to a compromised Q-factor due to the lossy substrate and the relatively low fill-factor of the frustum-shaped nanodisks. Furthermore, for both systems, the resonance behavior corresponding to the polarization conversion can be preserved upon optical excitations of the two discussed pump fluences. These observations indicate that the Q-factor of the in-plane eigenmode is not affected significantly by the increase in the imaginary part.


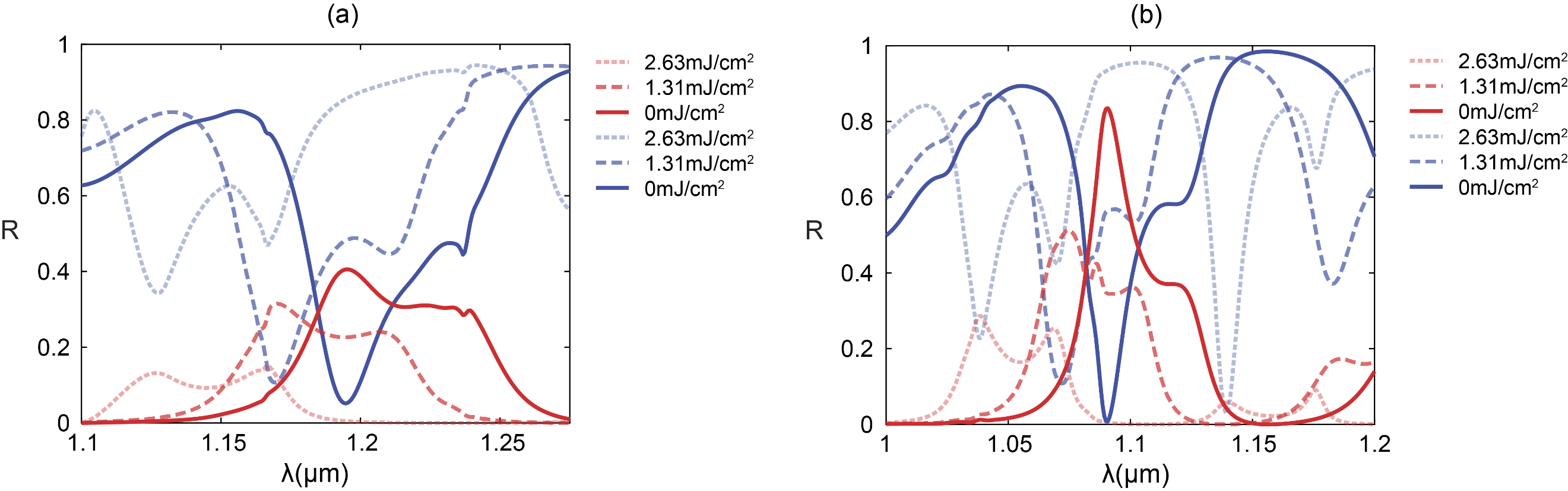


Fig. S2. Reflection spectra for two configurations of ZnO-based metasurfaces at pump fluences of 0mJ/cm^2^, 1.31mJ/cm^2^, and 2.63mJ/cm^2^. (a) R_ss_ (blue curve) and R_sp_ (red curve) for the ZnO/Sapphire/Ag metasurface (*a* = 1100nm, frustum-shaped nanodisk) at (*k_x_*, *k_y_*) = (0.06, 0.115)×*2πc/a*. (b) R_ss_ (blue curve) and R_sp_ (red curve) for the ZnO/PEC metasurface (*a* = 1000nm, cylinder-shaped nanodisk) at (*k_x_*, *k_y_*) = (0.04, 0.08)×*2πc/a*.

For the ZnO/Ag hybrid metasurfaces (same design discussed in the main text), to visualize the topological properties of the scattering field at 1197nm, the vector flow of the complex scattering coefficients, ((Re(r_ss_), Im(r_ss_)), are plotted on top of the reflectance (R_ss_). Corresponding to the polarization ellipse plots, Fig. 2(a)(b)(c) in main text, the *k-*space maps are provided under three pump fluences. A pair of topological charges of opposite winding numbers are observed on the map that are symmetrically located with respect to the ΓM. The rapidly varied polarization states across the momentum space, shown on the Fig. 2(a)-(c), are closely related to the topological properties of the vector scattering field. With the huge tunability offered by ZnO, a clear dynamic in the topological charges can be observed in the vector scattering field mapping. As the pump fluence gradually increase from 0 mJ/cm^2^ to 2.63 mJ/cm^2^, the positive and negative charges undergo a drift towards the ΓM direction in the *k-*space and eventually annihilate. Along with the drift of the topological charges, the polarization abundant domain and the on-resonance iso-frequency contour vary accordingly, indicating the topological origin of the polarization modulation. Second, as derived in Section S1 of the Supplementary Information, the topological charges of the scattering field and the winding SOPs along the on-resonance iso-frequency contours closely correlate with the rotation of the in-plane modes around the SOP singularity at the Γ point. Therefore, the polarization manipulation potential offered by the proposed metasurfaces originates from their topological properties.


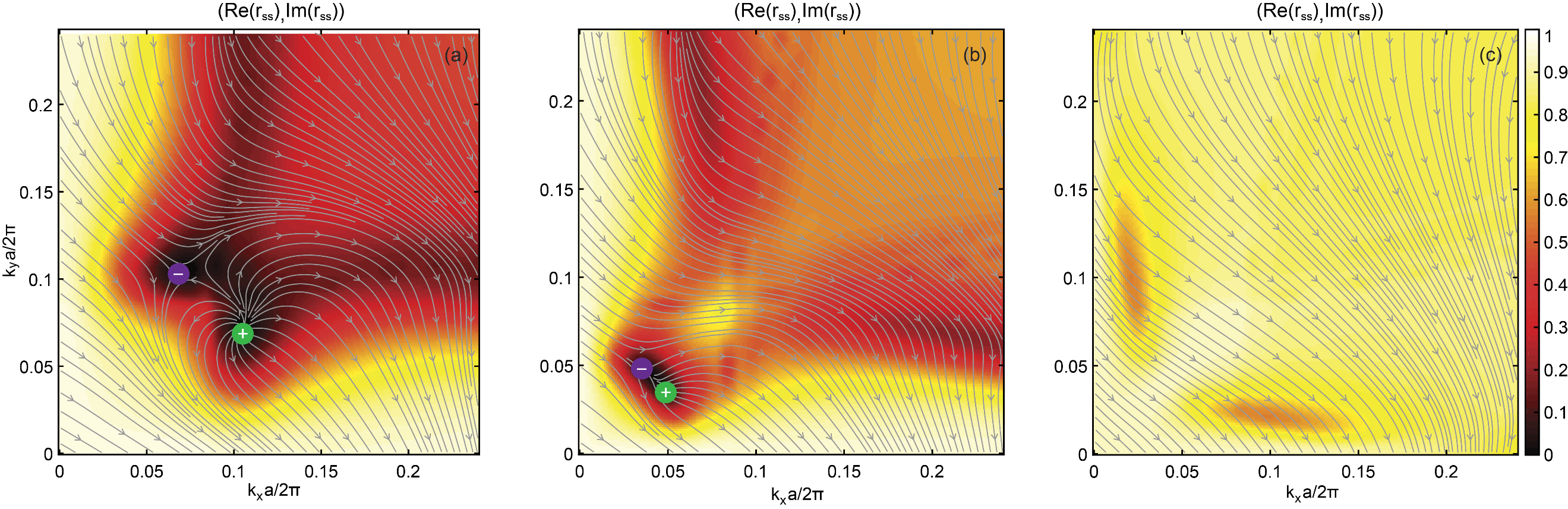


Fig. S3. Vector flow of the complex scattering coefficients, ((*Re*(*r_ss_*), *Im*(*r_ss_*)), on top of the reflectance, *R_ss_*, at *ω_0_* = 0.919×2π*c/a* (*λ*_0_ = 1197 nm) upon three pump fluence (a) 0 mJ/cm^2^, (b) 1.31 mJ/cm^2^, and (c) 2.63 mJ/cm^2^.

**S3. Complete polarization conversion in C_3_-symmetrical metasurfaces**

To better illustrate the potential of ZnO-based metasurfaces for polarization control, we further study a system with C_3_-rotational symmetry. In particular, the metasurface is composed of an array of cylinder-shaped ZnO nanodisks (radius *r* = 400 nm and height *h* = 200 nm) on top of a PEC substrate. The nanodisks are arranged in a honeycomb lattice with a lattice constant *a =* 1000 nm. The calculated band diagram is provided in Fig. S4(a). Fig. S4(b) and (c) show the corresponding mode profiles for a few bands. Similar to our observations in the C_4_-metasurfaces, along the ΓM and ΓK directions, the eigenmodes on different bands exhibit either odd-mode or even-mode characteristics due to the corresponding symmetry properties of the system.


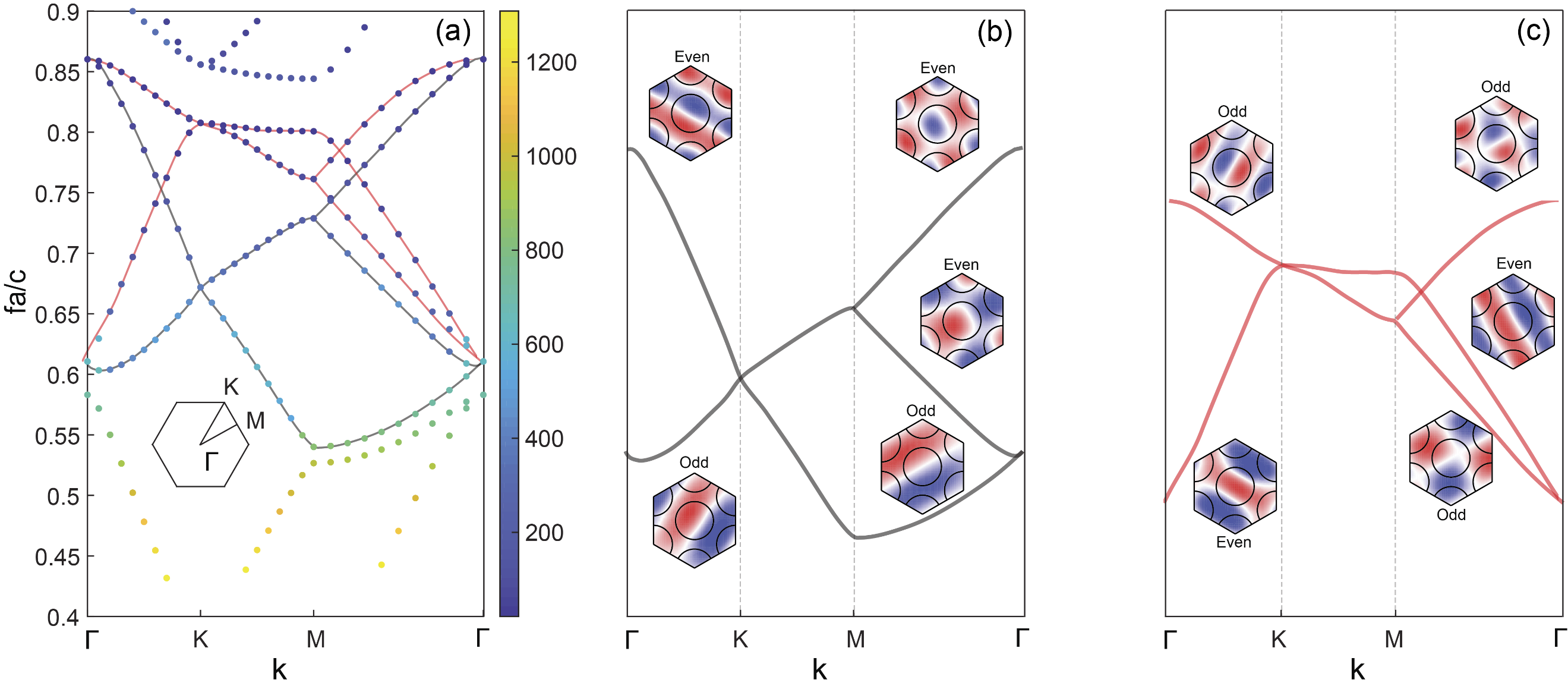


Fig. S4. ZnO/PEC hybrid metasurfaces with C_3_-rotational symmetry. (a) Band diagram for the hybrid metasurface consisting of a ZnO nanodisk array on top of a PEC backplane. The TE band with in-plane wavevector *k_||_* parallel to the ΓM, MK, ΓK directions are highlighted by the grey and red curves. The color-coded markers represent the corresponding radiative *Q*-factor. The ZnO nanodisk's radius (*r*) and height (*h*) are *r* =200 nm and *h* = 200 nm, respectively, while the lattice constant *a* = 1000 nm. The corresponding mode profiles of *E_z_* are shown in (b) and (c).

According to the analysis presented in previous studies (ACS Photonics **7**, 2362 (2020) and Phys. Rev. Lett. **119**, 167401 (2017)), the complete polarization conversion is expected to occur at an intermediate mode between the even and the odd modes. Furthermore, this intermediate mode reveals itself as singularities of the vectorized scattering coefficients, which can be categorized as topological charges in the scattering field. Implementing the same methodology, the intermediate modes can also be identified in the C_3_-metasurfaces. As shown in Fig. S5(a), a quasi-complete polarization conversion effect with an efficiency of up to 80% can be observed at *λ_0_* = 1271 nm for the incident wavevector (*k_x_*, *k_y_*) = (0.144, 0.217)×*2πc/a*. Similarly, in Fig. S5(d), the polarization conversion effect for an efficiency up to 70% can be observed at *λ_0_* = 1294 nm for the incident wavevector (*k_x_*, *k_y_*) = (0.147, 0.251)×*2πc/a*. At these two wavelengths, the polarization synthesized adjacent to the conversion wavevector in *k-*space is plotted as polarization ellipses in Figs. S5(b) and (e). The iso-frequency contours of the quasi-linear polarized scattering field can be seen in these polarization ellipse maps. We note that these iso-frequency contours are of similar winding nature as seen in the C_4_-metasurfaces but with higher order, manifesting the topological properties of the in-plane eigenmodes as discussed in Section S1 of the Supplementary Information. Meanwhile, a domain of rapid polarization variation can be identified on both sides of the iso-frequency contours, indicating the potential of the proposed C_4_-metasurface for active polarization modulation. In particular, a phase transition from a right-handed (RH)-polarized domain to a left-handed (LH)-polarized domain occurs at the iso-frequency contour, as predicted in Section S1 of the Supplementary Information. Furthermore, these domains are located symmetrically with respect to the ΓM and ΓK directions. In both Fig. S5(c) and (f), two singularities (green and purple dots) with opposite winding numbers are symmetrically distributed with respect to the ΓM direction in the vector field plots (*i.e.*, ($\mathfrak{R(}r_{ss}\mathfrak{), I(}r_{ss})$). Due to the C_3_-rotational symmetry, another topological charge of a positive winding number is present in the first quadrant, a mirror reflection of the negative charge (purple dot) with respect to the ΓK direction. In other words, for the C_3_-symmetric system, there exist three scattering topological charges in the first quadrant. These observations confirm the highly diverse polarization properties offered by C_3_-metasurfaces in momentum space and suggest potential applications for ZnO-based systems in active and large-scale polarization control based on their topology properties.


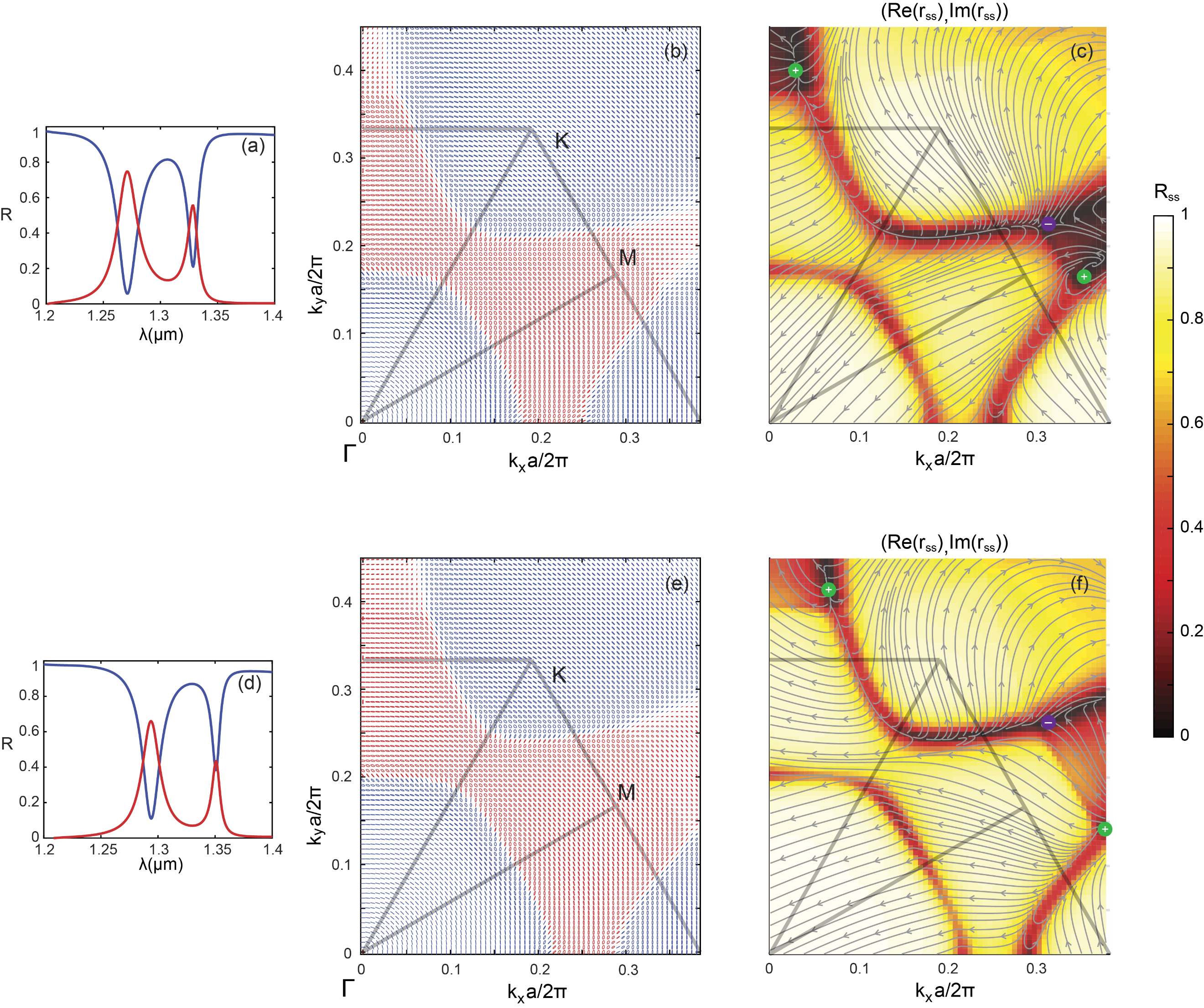


Fig. S5. Scattering properties of a ZnO/PEC hybrid metasurface with C_3_-rotational symmetry. (a) Reflection spectrum at a wavevector of (*k_x_*, *k_y_*) = (0.144, 0.217)×*2πc/a*. (b) Polarization ellipses as a function of wavevectors for *s*-pol incident light at *ω_0_* = 0.787×2π*c/a* (*λ*_0_ = 1271 nm). (c) Vector flow of the complex scattering coefficients, ((*Re*(*r_ss_*), *Im*(*r_ss_*)), superimposed on the reflectance, *R_ss_*, at *ω_0_* = 0.787×2π*c/a* (*λ*_0_ = 1271 nm). (d) Same as panel (a) but for a wavevector of (*k_x_*, *k_y_*) = (0.147, 0.251)×*2πc/a*. (e) Same as panel (b) but for *ω_0_* = 0.773×2π*c/a* (*λ*_0_ = 1294 nm). (f) Same as panel (c) but for *ω_0_* = 0.773×2π*c/a* (*λ*_0_ = 1294 nm).

**References:**

(1) Fan, S.; Suh, W.; Joannopoulos, J. D. Temporal Coupled-Mode Theory for the Fano Resonance in Optical Resonators. *J. Opt. Soc. Am. A* **2003**, *20* (3), 569. https://doi.org/10.1364/JOSAA.20.000569.

(2) Guo, Y.; Xiao, M.; Fan, S. Topologically Protected Complete Polarization Conversion. *Phys. Rev. Lett.* **2017**, *119* (16), 167401. https://doi.org/10.1103/PhysRevLett.119.167401.

(3) Wu, Y.; Kang, L.; Bao, H.; Werner, D. H. Exploiting Topological Properties of Mie-Resonance-Based Hybrid Metasurfaces for Ultrafast Switching of Light Polarization. *ACS Photonics* **2020**, *7* (9), 2362–2373. https://doi.org/10.1021/acsphotonics.0c00858.
